# Unveiling Genetic Potential for Equine Meat Production: A Bioinformatics Approach

**DOI:** 10.3390/ani14162441

**Published:** 2024-08-22

**Authors:** Martin Šimon, Ana Kaić, Klemen Potočnik

**Affiliations:** 1 Department of Animal Science, Biotechnical Faculty, University of Ljubljana, Groblje 3, 1230 Domžale, Slovenia; martin.simon@bf.uni-lj.si (M.Š.); klemen.potocnik@bf.uni-lj.si (K.P.); 2 Department of Animal Science and Technology, Faculty of Agriculture, University of Zagreb, Svetošimunska 25, 10000 Zagreb, Croatia

**Keywords:** bioinformatics, equine genomics, horsemeat production, selective breeding, SNP markers

## Abstract

**Simple Summary:**

As global meat production is rapidly increasing, it is important to find sustainable sources of meat. Horsemeat is a viable alternative due to its lower environmental impact and high nutritional value. In this study, bioinformatics was used to determine genetic markers (SNPs) that could improve horsemeat production by focusing on orthologous genes related to meat yield in cattle and in QTLs for body weight and body size. Out of 268 markers, 27 SNPs in key genes such as *LCORL*, *LASP1*, *IGF1R*, and *MSTN* were prioritized. These findings can help small-scale farmers to breed horses with better meat yield. In addition, this information will be valuable for large-scale genetic studies to further evaluate these markers and improve breeding programs. This work lays the foundation for a better understanding of horse genetics and the advancement of horse breeding.

**Abstract:**

In view of the predicted significant increase in global meat production, alternative sources such as horsemeat are becoming increasingly important due to their lower environmental impact and high nutritional value. This study aimed to identify SNP markers on the GeneSeek^®^ Genomic Profiler™ Equine (Neogen, Lansing, MI, USA) that are important for horsemeat production traits. First, orthologous genes related to meat yield in cattle and common genes between horses and cattle within QTLs for body size and weight were identified. Markers for these genes were then evaluated based on predicted variant consequences, GERP scores, and positions within constrained elements and orthologous regulatory regions in pigs. A total of 268 markers in 57 genes related to meat production were analyzed. This resulted in 27 prioritized SNP markers in 22 genes, including notable markers in *LCORL*, *LASP1*, *IGF1R*, and *MSTN*. These results will benefit smallholder farmers by providing genetic insights for selective breeding that could improve meat yield. This study also supports future large-scale genetic analyses such as GWAS and Genomic Best Linear Unbiased Prediction (GBLUP). The results of this study may be helpful in improving the accuracy of genomic breeding values. However, limitations include reliance on bioinformatics without experimental validation. Future research can validate these markers and consider a wider range of traits to ensure accuracy in equine breeding.

## 1. Introduction

**Figure 1:**
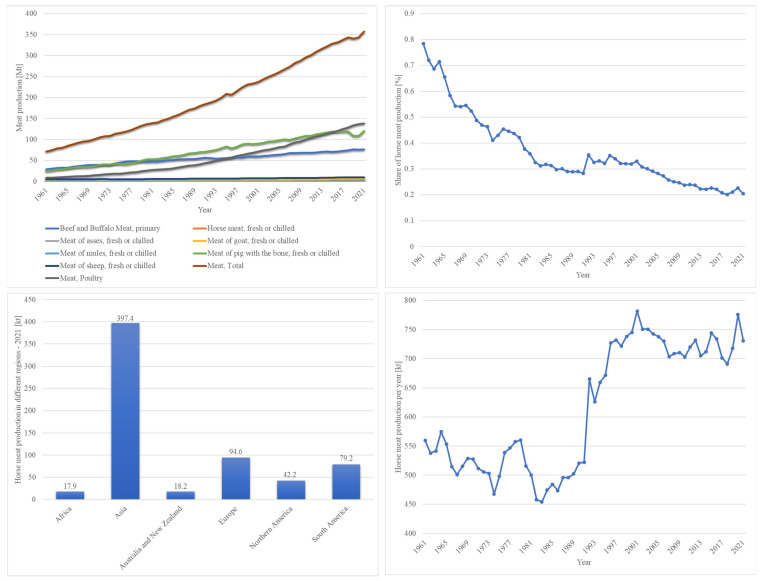
Trends and statistics in global meat production (1961–2021). Top Left: Total global meat production by type (in million tons) from 1961 to 2021. Top Right: Share of horse meat in total meat production (in percentage) from 1961 to 2021, showing a declining trend over the years. Bottom Left: Horse meat production by region (in million tons) in 2021, highlighting Asia as the leading producer. Bottom Right: Annual global horse meat production (in thousand tons) from 1961 to 2021, illustrating fluctuations and an overall increasing trend in recent years [2].

With global meat production exceeding 350 million tons in 2021 and projected to rise to 460 to 570 million tons by 2050 [1,2], the urgency of finding sustainable sources of meat is clear. Despite its modest share in the global market and a stagnating production trend since 1997 [2] (Figure 1), horsemeat represents a viable alternative, particularly in light of the environmental challenges posed by resource-intensive meat production methods [2,3,4]. Horsemeat production is characterized by lower methane emissions due to the horse’s unique digestive system [5].

It is estimated that the per capita supply of horsemeat for 2021 (calculated as total production plus imports minus exports divided by population size) is approximately 0.10 kg per person worldwide. Certain countries are well above this global average, including Mongolia (17.15 kg), Kazakhstan (8.00 kg), Kyrgyzstan (4.28 kg), Uruguay (1.54 kg), Iceland (1.43 kg), Australia (0.97 kg), Mexico (0.58 kg), Chile (0.54 kg), Canada (0.48 kg), Haiti (0.43 kg), Belgium (0.42 kg), Senegal (0.42 kg), Italy (0.42 kg), Russia (0.36 kg), Argentina (0.33 kg), Switzerland (0.28 kg), Nicaragua (0.28 kg), and Finland and Paraguay (0.22 kg, each) [2]. All of these countries, with the exception of Belgium, Italy, and Switzerland, are also among the leading per capita producers of horsemeat. These figures illustrate the considerable regional differences in the production and consumption of horsemeat and highlight the generally low global consumption of this resource.

The nutritional composition of horsemeat, which is significantly lower in fat and cholesterol and richer in n-3 fatty acids and heme iron compared to pork, beef, or poultry, shows that it has the potential to improve human health by improving lipid profiles and iron status [6,7]. Its unique fatty acid composition, particularly its high polyunsaturated fatty acid (PUFAs) content, not only offers a healthier alternative to traditionally consumed red meat but also suggests its role in the dietary management of disease. The higher content of palmitoleic and alpha-linolenic acids compared to beef and pork could protect against cardiovascular disease, stroke, and neurodegeneration [8]. In addition, the high content of high-quality proteins, essential amino acids, vitamins, and minerals suggests that horsemeat should be included in the diet of patients with metabolic obesity, as it offers potential therapeutic benefits in weight control and improving liver function [9]. The promising results on horsemeat hydrolysates suggest possible protection against muscle atrophy [10]. Overall, the nutritional benefits of horsemeat contribute significantly to its potential as a valuable dietary component for the prevention and treatment of various health conditions.

In Spain, where 15.3% of horse farms are dedicated to meat production, the traditional rearing of native breeds for meat production emphasizes the potential to conserve genetic diversity and native and/or endangered breeds, as well as to support the rural economy [11]. These practices contribute to the conservation of rural landscapes by providing important environmental services and preserving mosaic landscapes and biodiversity. In addition, extensive horse breeding helps to reduce the risk of forest fires, as less fuel is used for machinery [12]. The nutritional value of horsemeat and the environmental benefits of horsemeat production, including the contribution to the conservation of disadvantaged grazing lands, make horsemeat a viable solution to sustainability challenges in the meat industry [5,13,14]. In addition, the processing of horsemeat into various products such as “cecina”, dry-cured loin, and salami offers an attractive, gastronomically rich alternative to conventional meat products, promoting dietary diversification and preserving traditional culinary practices [15]. Additionally, Kołodziejczyk et al. (2019) highlight that horse breeding, especially cold-blooded horses, can be economically beneficial due to cheaper feed for horses, excellent feed conversion rates, rapid growth and weight gain, and high slaughter efficiency, making horse meat production potentially more profitable than other livestock such as cattle [16].

The traditional system of meat production is based on the rearing of native breeds that were formerly used as draft animals and were converted to meat production after the mechanization of agriculture [11]. The benefits of horsemeat production are overshadowed by the cultural view that horses are mainly bred for sport and leisure activities, which has resulted in few genomic studies on equine production traits compared to the extensive studies in cattle. Closing this gap will require overcoming cultural reservations and developing markets that are receptive to the benefits of horsemeat, underlining the urgency of further genomic research.

Beef production plays a central role in the global meat industry, supported by intensive research to improve various aspects of production, including nutrition, selective breeding, and genotyping. The quest for genetic improvement of cattle breeds depends on the identification of genetic markers associated with desirable traits. Key techniques such as genomic selection, genome-wide association studies (GWAS), and quantitative trait loci (QTL) mapping play an essential role in this process [17], facilitating the discovery of genetic variations associated with economically important traits such as feed efficiency and carcass quality. In addition, advances in genotyping of cattle populations enable the analysis of large cohorts, allowing accurate estimation of genomic breeding values for specific traits established in national breeding programs. These estimates are made possible by advanced genomic Best Linear Unbiased Prediction (BLUP) models, which significantly improve the accuracy and efficiency of breeding strategies [18].

**Figure 2:**
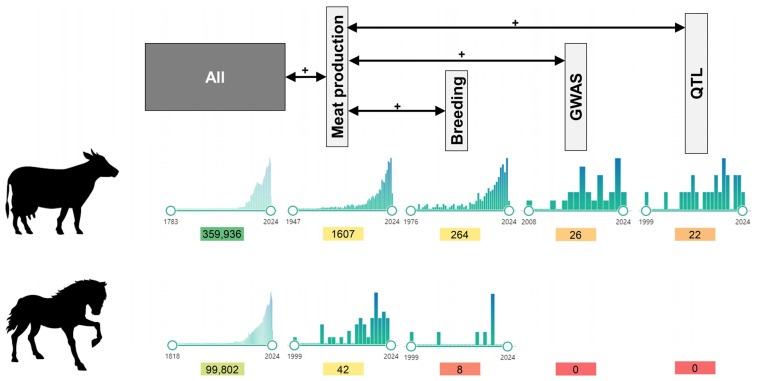
Comparative analysis of genetic research in cattle and horses for meat production (1783–2024).

Conversely, efforts to genotype horses, particularly concerning their production potential, have been neglected (Figure 2). Moreover, the most comprehensive publicly available database on QTLs and single-nucleotide polymorphism/gene association data of livestock species does not contain QTLs or associated genes/markers related to equine production traits.

To address this gap, we conducted a comparative genomic analysis with cattle, which have significant physiological and genetic similarities to horses, particularly with respect to traits such as body size and weight that are critical for meat production. Cattle have a wealth of well-annotated genomic and QTL data, making them a robust model for identifying relevant genes and markers in horses. Although pigs are another important livestock species, they are less suitable as a model for horses due to their different dietary requirements and housing systems. Pigs are omnivores and are typically kept in intensive production systems, which differ significantly from the extensive grazing systems used for horses. Sheep and goats, on the other hand, have more similarities with horses in terms of grazing behavior and certain physiological characteristics. However, there is relatively little research on these species compared to cattle, making them less suitable as primary models. Therefore, this study aims to apply the knowledge gained in cattle (meat production-associated QTLs) to horses by prioritizing the markers on the GGP Equine chip. This approach could help horse breeders in their decision-making processes. In addition, the results presented here could later be enriched with data from GWAS and other genomic evaluations performed on larger horse populations to provide the basis for future advances.

## 2. Materials and Methods

### 2.1. Literature Search

On 29 March 2024, a systematic search was conducted in the PubMed database (https://pubmed.ncbi.nlm.nih.gov/ (accessed on 29 March 2024)) to collate research about the production of horse and cattle meat, looking in particular for literature on genome-wide association studies (GWAS), quantitative trait loci (QTL), and breeding strategies. The search terms and the strategy used for this inquiry are listed in Table S1.

### 2.2. Genes Related to Meat Production Retrieval

QTLs related to meat growth/size/meat production in cattle and horses were acquired from the Animal QTLdb (Release 52, https://www.animalgenome.org/cgi-bin/QTLdb/index (accessed on 10 February 2024)) [19]. For QTLs spanning broad genomic regions, genes in cattle were initially identified through g:Profiler (https://biit.cs.ut.ee/gprofiler/gost (accessed on 15 February 2024)) [20], followed by determination of their orthologous counterparts in horses. In cases where the database provided only peak QTL positions, the Variant Effect Predictor (VEP) (Version 111, https://www.ensembl.org/Tools/VEP (accessed on 16 February 2024)) [21] was utilized to expand the search to include genes with variants within a range of 5000 bp upstream and downstream, as this interval is likely to capture relevant regulatory elements that may be located near the gene. Subsequently, the orthologous genes were identified in horses.

### 2.3. Identification of SNP Markers on the GGP Equine

The Ensembl BioMart tool (https://www.ensembl.org/biomart/martview/ (accessed on 17 February 2024)) [22] was used to determine the genomic coordinates of candidate genes in horses (EquCab3.0). To capture potential regulatory variants, an interval of 5000 bp was subtracted from the start and added to the end of each gene’s coordinates. This extended range ensures the inclusion of variants located in the potential regulatory regions upstream and downstream of the genes. Markers within the above regions were then extracted from the GeneSeek^®^ Genomic Profiler™ Equine, which contains markers for over 70,000 SNPs. These markers were then evaluated using the bioinformatics tools described below.

### 2.4. Bioinformatics Analyses

The Variant Effect Predictor (VEP) (version 111, https://www.ensembl.org/Tools/VEP (accessed on 16 February 2024)) [21] was utilized to determine the predicted consequences of variants for the selected markers. Additionally, the GERP score, defined as the reduction in the number of substitutions in the multi-species sequence alignment compared to the neutral expectation [23], was obtained for each of the markers, as well as their potential location within constrained element and regulatory elements at orthologous sites in pigs, using the Ensembl database (Version 111, https://www.ensembl.org/index.html (accessed on 25 February 2024)) [22].

To assess the biological significance of the identified genes, the STRING database (Version 12.0, https://string-db.org (accessed on 5 March 2024)) [24] was used to explore potential protein–protein interactions and to extract gene ontology and Kyoto Encyclopedia of Genes and Genomes (KEGG) pathway annotations. Additionally, parental KEGG orthology terms were extracted from the KEGG database (Release 109, https://www.genome.jp/kegg (accessed on 21 March 2024)) [25] to better understand the functional roles of these genes. Gene associations with mammalian phenotypes were obtained from the Mouse Genome Database (MGD, 2024, https://www.informatics.jax.org (accessed on 2 April 2024) [26], providing insights into potential phenotypic effects. Cytoscape (Version 3.10.1) was used for the visualization [27]. The potential involvement of candidate SNPs within selected genes in meat production was validated by (1) comparing alleles between one of the largest horse breeds, Percheron (P), and one of the smallest, American Miniature (AM), and (2) comparing protein sequence similarities for the genes with the SNPs that differ between the two breeds. For allele comparison, each of the prioritized SNPs was searched in the Ensembl database, and sample genotypes were obtained. For sequence similarity analysis, orthologous genes associated with high-priority candidate SNPs were identified using the Ensembl database, which also provided protein sequence similarity data.

## 3. Results

**Figure 3:**
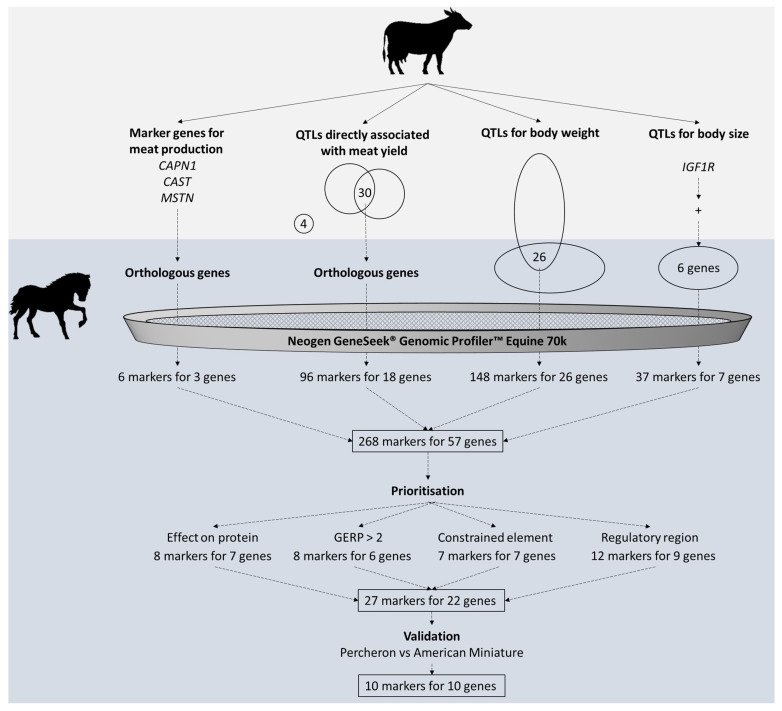
Workflow for identifying and prioritizing SNP markers for horsemeat production traits. Note: The markers in this figure represent unique SNP locations, excluding duplicate SNP probes.

In this study, we focused on the identification of SNP markers on the GGP Equine chip that are potentially important for horsemeat production traits. We began by identifying orthologous genes critical for meat production in cattle, followed by the identification of common genes within QTLs for body size and body weight. Next, we obtained markers within these regions from the GeneSeek^®^ Genomic Profiler™ (GGP) Equine (Neogen, Lansing, MI, USA), which contains over 70,000 SNP markers. A total of 278 markers (Table S2) in 57 genes were identified. It is important to note that several SNP markers target the same genomic locations, resulting in duplicates: BIEC2_806771 and BIEC2_806771_DUP map to 3:101540587; 3-102641557-C-T and 3-102641557-C-T_DUP map to 3:102641557; CUHSNP00061902 and UKUL845 map to 3:103779615; BIEC681989 and BIEC681989_DUP map to 3:104518656; BIEC2_808543, GGP_100_BODY_SIZE_ECA3_F, and GGP_100_BODY_SIZE_ECA3_F_2 all map to 3:107374136; GGP_103_BODY_SIZE_ECA11_F and GGP_103_BODY_SIZE_ECA11_F_2 map to 11:23334511; BIEC2_438999, GGP_099_RACING_DISTANCE_MSTN_BIEC2-438999_F, and GGP_099_RACING_DISTANCE_MSTN_BIEC2-438999_F_2 all map to 18:67027762. The unique 268 positions were then evaluated considering the predicted variant consequences, GERP scores, and their positions within constrained elements and orthologous regulatory regions in pigs. This analysis resulted in 27 prioritized SNP markers for 22 genes. The validation of these markers by comparing the genotypes of one of the largest and one of the smallest horse breeds, Percheron (P) and American Miniature (AM), revealed 10 strong SNP candidate markers for 10 genes (Figure 3).

### 3.1. Comparative Analysis of SNP Markers on Bovine and Equine Chips

We initiated our investigation by identifying SNP markers on the GGP Equine chip within (and 5000 bp up- and down-stream) *CAPN1*, *CAST*, and *MSTN*, frontline genes directly associated with meat yield and quality in cows.

For *CAPN1*, GGP Equine contains a marker (CUHSNP00133513) for the SNP rs396701927. The SNP is a missense variant. Due to its high GERP score (4.15) and location within a constrained element, the SNP rs396701927 is an interesting candidate for horsemeat production. Two markers, BIEC2_277089 and BIEC2_277118, are for SNPs rs68930623 and rs68930652 within *CAST*, intronic variants of the canonical transcript ENSECAT00000052865. The rs68930652 is also an upstream variant of the ENSECAT00000031937. It is worth mentioning that the orthologous location of rs68930623 in pigs is within the enhancer. GGP Equine contains two SNP markers for *MSTN*, BIEC2_417365 (SNP rs69126368) and BIEC2_417369 (SNP rs69126372), which target intronic regions. The rs69126368 is located within a constrained element, potentially being an important marker in horses. In addition, there is a marker called GGP_099_RACING_DISTANCE_MSTN_BIEC2_438999 on the GGP Equine chip targeting rs69127737 within *NEMP2* but with a low GERP score and also not within an orthologous regulatory element (Table S3).

### 3.2. QTLs Directly Associated with Meat Production in Cattle

No “meat”-associated QTLs have yet been described in the Animal QTLdb for horses. CattleQTLdb groups 19 QTLs associated with meat production into 3 categories: dressed carcass muscle percentage, dressed carcass muscle weight, and dressed carcass muscle-to-bone ratio (Table S4).

The QTLs for dressed carcass muscle percentage contain 499 genes, dressed carcass muscle weight 4 genes, and dressed carcass muscle-to-bone ratio 516 genes. While none of the genes were associated with all 3 groups, 30 were associated with 2, all of them with dressed carcass muscle weight and dressed carcass muscle-to-bone ratio: *ADGRA3*, *ANAPC4*, *bta-mir-218-1*, *CCDC149*, *CCKAR*, *DHX15*, *ENSBTAG00000018885*, *ENSBTAG00000033327*, *ENSBTAG00000049001*, *GBA3*, *KCNIP4*, *LGI2*, *PACRGL*, *PCDH7*, *PI4K2B*, *PPARGC1A*, *RBPJ*, *SEL1L3*, *SEPSECS*, *SLC34A2*, *SLIT2*, *SMIM20*, *SNORA70*, *SOD3*, *STIM2*, *TBC1D19*, three genes coding for *U6*, and *ZCCHC4*.

**Figure 4:**
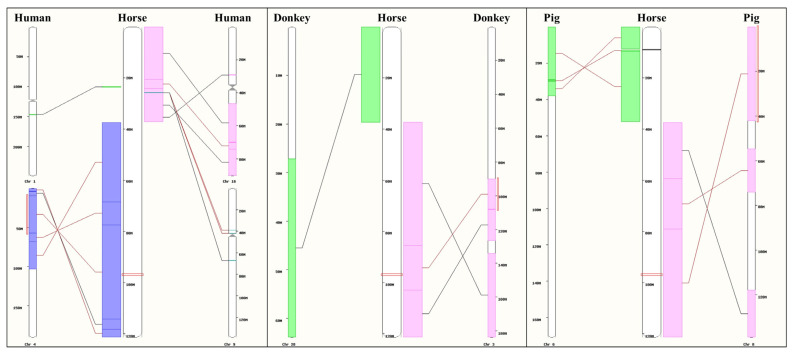
Syntenic block on chromosome 3 in horses. Red rectangles on the horse chromosome indicate the region of orthologous genes associated with dressed carcass muscle weight and dressed carcass muscle-to-bone ratio in cattle.

For these 30 genes, 22 horse orthologous genes were retrieved, all located on chromosome 3, in a syntenic block (Figure 4) rich in horse growth-related QTLs, such as body weight, size, and chest circumference. In total, 96 SNP markers for 18 genes are present on the GGP Equine chip (Table S5).

Most markers may target regions that influence transcription. Of particular note is a 3 prime UTR variant (rs1147560021) in *LGI2* with a GERP score of 2.7. This variant may alter the miRNA binding site. Another variant is the intron variant rs68672779 in *PPARGC1A* with a GERP score of 3.57. In addition, the orthologous location of a 5 prime UTR variant rs68670656 and an upstream gene variant rs68508249 of *SEPSECS* and *ADGRA3* are located within the promoter and enhancer, respectively, in pigs. Variants that may alter protein sequence and function include two missense variants rs68555658 and rs1147724321 (markers UKUL834 and UKUL835) in *PCDH7* with GERP scores 2.83 and 3.84, respectively, a stop-lost variant rs1141209077 (marker CUHSNP00004551) in *PI4K2B*, and a splice donor region variant rs68593800 (marker CUHSNP00004551) in *SLIT2*, located in constrained element.

### 3.3. Analysis of QTLs Indirectly Involved in Meat Production

There are 35 QTLs associated with production in cows, but none in horses. However, when examining QTLs associated with horse growth, there are three main traits in common related to production: body weight, body length, and (overall) body size. In the following subsections, we focused on body weight and body size.

#### 3.3.1. Body Weight

There are 1135 genes within the body weight QTLs in cows, but only 26 in horses, 2 of which are common to both species, *ITPRID2* and *FAM184B*. The remaining 24 genes in horses are as follows: *ABI2*, *C18H2orf88*, *COL5A2*, *DNAH7*, *RAPH1*, *ENSECAG00000033069*, *ENSECAG00000043662*, *ENSECAG00000043877*, *ENSECAG00000046295*, *ENSECAG00000046532*, *ENSECAG00000052525*, *ENSECAG00000053791*, *ENSECAG00000057875*, *GULP1*, *LDB2*, *MED28*, *MFSD6*, *MYO1B*, *NAB1*, *NEMP2*, *SLC39A10*, *TFPI*, and *ZNF804A*.

For the genes in horses, we obtained SNP markers on the GGP Equine chip. There are 148 markers for the above genes, including the markers of GGP_099_RACING_DISTANCE_MSTN_BIEC2-438999 and GGP_100_BODY_SIZE_ECA3 for body size mentioned above. The summarized table of the variant consequences of the body weight markers is given in Table S6.

Of note are three missense variants in *DNAH7* (rs396935555), *ZNF804A* (rs69192315), and *GULP1* (18_65173441_T/C). A not-yet-reported variant of *GULP1* is also a 5 prime UTR variant of another transcript. Its high GERP score (4, 81) and the location within a constrained element make this SNP a likely candidate. High GERP scores are also for SNPs rs68525653 (3.0) and 18_65288583_A/C (3.2), located in an intron of *LDB2* and in the 3 prime UTR of *GULP1*, respectively. Four SNPs are within the orthologous enhancer: rs68454110 and rs68534807 in *FAM184B*, rs68603064 in *ENSECAG00000052525* (GGP_100_BODY_SIZE_ECA3), and rs68525607 in *LDB2*. Other SNPs located in constrained elements, although having low GERP scores, are rs69126368 in *C18H2orf88* and *MSTN* and rs69153418 and rs69153424 in *ABI2* and *RAPH1*.

The STRING tool enriched these genes based on the bovine orthologs [28] as genes involved in muscularity: *GULP1*, *NAB1*, *MFSD6*, *MYO1B*, and *COL5A2*. Interestingly, *MFSD6*, *NAB1*, and *MSTN* are in strong linkage disequilibrium in mammals [29].

#### 3.3.2. Body Size

There are only three QTLs for body size in cattle. Two of them are intergenic regions, and a QTL 57,073 overlaps with only one gene, *IGF1R*. In contrast, 12 QTLs were mapped to body size in horses, which overlap with 6 genes: *LASP1*, *OXCT1*, *FAM184B*, *MED28*, *QDPR,* and *ENSECAG00000052525*. Here, it is worth emphasizing *ENSECAG00000052525*, *FAM184B*, and *MED28* that are in body weight QTL as well.

For the genes in horses, we obtained SNP markers on the GGP Equine chip. There are 37 unique targets for the above genes, including the “cow” gene *IGF1R*. There are also two markers for body size on the chip, GGP_100_BODY_SIZE_ECA3 for lncRNA *ENSECAG00000052525* and GGP_103_BODY_SIZE_ECA11 (intron of *LASP1*) (Table S7).

Here, we would like to mention potential regulatory variants, with the exception of those mentioned above for *ENSECAG00000052525* and *FAM184B*. Interestingly, three SNPs within *LASP1* are in the orthologous enhancer: rs68875002, rs68876315, and a marker for body size rs68876319 (GGP_103_BODY_SIZE_ECA11). While rs68520444 is also localized in the enhancer of *QDPR*, rs68514854 is within the orthologous open chromatin region of *IGF1R*.

### 3.4. Gene Annotations

#### 3.4.1. Protein–Protein Interaction Networks and Biological Pathway of Candidate Genes for Horsemeat Production

**Figure 5:**
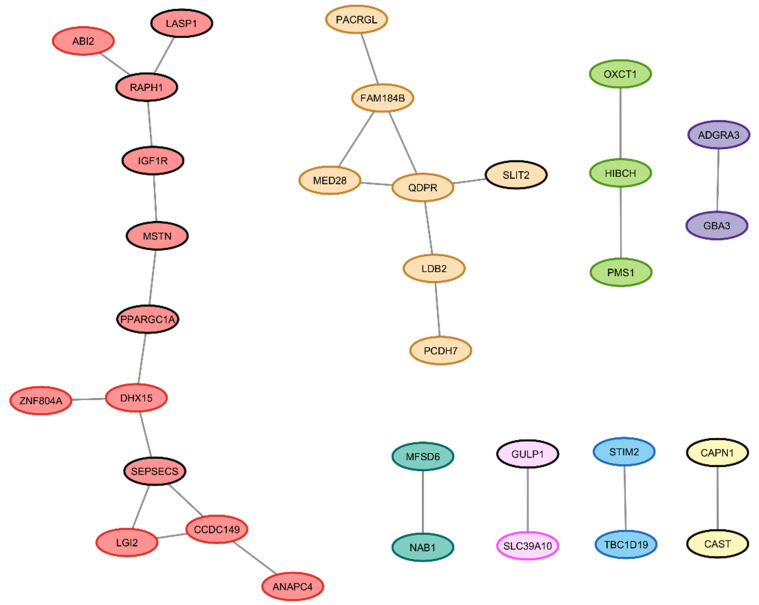
Protein–protein interaction (PPI) network of 43 candidate genes for horsemeat production analyzed using the STRING tool. Nodes circled in black indicate candidate genes with prioritized markers.

Of the 57 candidate genes, 43 were successfully imported into the STRING tool, with 27 edges among them. Compared to the expected five edges, the PPI enrichment was significant, with a *p*-value of 5.31 × 10^−11^. In total, 2 major PPI clusters were identified, consisting of 12 and 7 proteins, respectively. Interestingly, 6 genes (out of 12) with prioritized SNPs are included in the larger of the 2 clusters (Figure 5).

**Figure 6:**
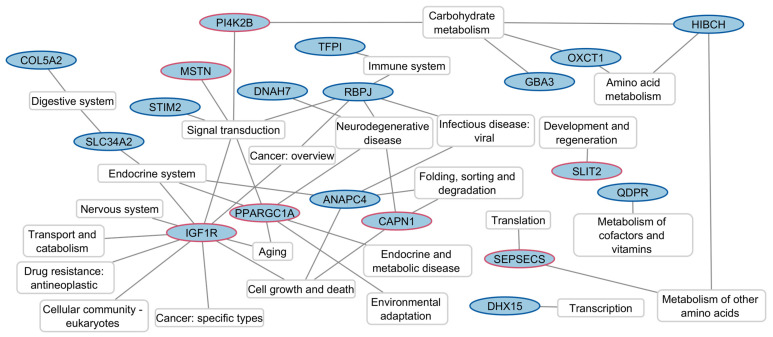
KEGG pathway analysis of 19 candidate genes for horsemeat production analyzed using the KEGG database. Nodes circled in red indicate candidate genes with prioritized markers.

While no single gene ontology term was enriched, 19 genes are annotated in the KEGG database. Analysis of the KEGG pathways reveals that the genes studied are predominantly involved in signal transduction pathways that are central to the regulation of growth, nutrient sensing, and energy homeostasis (*ANAPC4*, *IGF1R*, *CAPN1*, *MSTN*, *PI4K2B*, *PPARGC1A*, *RBPJ*, *STIM2*). Associated with this pathway, a significant subset is implicated in the endocrine system, highlighting roles in reproductive function, hormone signaling, and calcium regulation (*ANAPC4*, *IGF1R*, *PPARGC1A*, *SLC34A2*). Additionally, a notable group is involved in carbohydrate metabolism, emphasizing their contribution to energy production and management in the organism (*GBA3*, *HIBCH*, *OXCT1*, *PI4K2B*). Other important pathways include nutrient absorption (*COL5A2*, *SLC34A2*), transcription (*DHX15*), translation (*SEPSECS*), amino acid metabolism (*HIBCH*, *SEPSECS*), and protein turnover (*ANAPC4*, *CAPN1*). In addition, signaling pathways related to the nervous system (*IGF1R*, *CAPN1*, *DNAH7*, *RBPJ*, *PPARGC1A*) and the immune system (*RBPJ*, *TFPI*) were detected (Figure 6).

#### 3.4.2. Mammalian Phenotype

**Figure 7:**
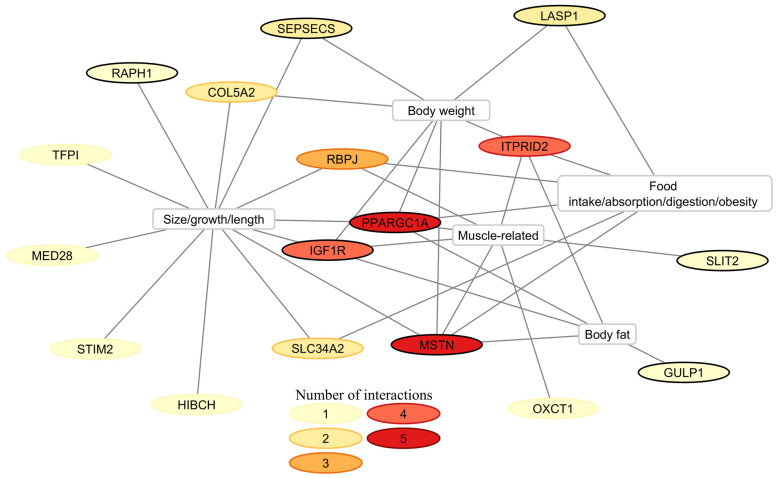
Mammalian phenotype associations of 18 genes from the study. These genes are linked to various traits possibly important for horsemeat production. Nodes circled in black indicate candidate genes with prioritized markers.

Subsequently, 18 genes (of the aforementioned 57) were identified that are associated with mammalian phenotypes related to body weight, growth, size, body fat, muscle development, feed uptake, and absorption. Twelve of these genes are associated with body size/growth/length. Also of interest are six genes related to feed/nutrient absorption and digestion. Perhaps more importantly, *Ppargc1a* and *Mstn* are associated with all five trait groups, while *Igf1r* and *Itprid2* are associated with four (Figure 7). Of the 22 genes for which SNPs were prioritized, 8 are associated with screened mammalian phenotypes: *GULP1*, *IGF1R*, *LASP1*, *MSTN*, *PPARGC1A*, *RAPH1*, *SEPSECS*, and *SLIT2* (Figure 7).

Table 1 summarizes the prioritized SNP markers and the corresponding genes, focusing on the reasons for their prioritization. The markers were selected based on potential effects on protein function, GERP scores greater than 2, their presence within constrained elements, and their location within orthologous regulatory elements. Notably, the gene *GULP1* has SNP markers with potential effects on protein function, high GERP scores, and is located within constrained elements, emphasizing its importance in the study. Other genes such as *CAPN1*, *MSTN*, and *SLIT2* also show multiple criteria for prioritization, underscoring their potential significance in horsemeat production traits.

Finally, the potential involvement of the prioritized SNPs in meat production in horses was validated by comparing alleles between one of the largest and one of the smallest horse breeds, P and AM. The analysis revealed ten SNPs in ten genes that differ between the two breeds, including rs68603064 (*ENSECAG00000052525*/*LCORL*, P: C|C, AM: T|T), rs68454110 (*FAM184B*, P: C|C, AM: T|T), rs68514854 (*IGF1R*, P: G|A, AM: A|A), rs68875002 (*LASP1*, P: T|T, AM: C|C), rs69126368 (*C18H2orf88*/*MSTN*, P: A|G, AM: G|G), rs68555658 (*PCDH7*, P: T|G, AM: G|G), and rs68520444 (*QDPR*, P: A|A, AM: A|G), among others. These markers are marked in bold in Table 1, and the corresponding genotypes are listed in Table S8. To further validate the relevance of these SNPs, we compared the sequence similarities of the orthologous genes between horses and cattle. Proteins with more than 60% sequence similarity are likely to have similar functions [30]. Table S9 provides an orthologous gene comparison between horses and cattle, showing that all genes have sequence similarities above 60%, with many genes such as *IGF1R*, *LASP1*, and *MSTN* having identity percentages exceeding 94%.

## 4. Discussion

In this study, we focused on the identification of SNP markers on the GGP Equine chip, which contains over 70,000 SNP markers, potentially relevant to horsemeat production traits. We began by identifying orthologous genes related to meat yield in cattle and QTLs for body weight and body size. Using these identified regions, we obtained 268 markers for 57 genes from the chip. These 268 markers were then evaluated considering predicted variant consequences, GERP scores, and their positions within constrained elements and orthologous regulatory regions in pigs. The predicted variant effects help us to identify SNPs that may affect protein function or gene regulation. High GERP scores and positions within constrained elements suggest that these SNPs are in evolutionarily significant regions, indicating their crucial role in biological processes. In addition, SNPs that match orthologous regulatory elements in pigs might affect gene transcription.

Out of 268 SNP markers, 27 for 22 genes were prioritized, including 2 already known markers for body size, GGP_100_BODY_SIZE_ECA3 for rs68603064 in lncRNA *ENSECAG00000052525* and GGP_103_BODY_SIZE_ECA11 for rs68876319 in *LASP1*. However, the marker GGP_100_BODY_SIZE_ECA3 is usually assigned to the *LCORL*/*NCAPG* genes [31,32]. Both *LCORL* and *LASP1* are involved in osteogenesis [33]. The rs68603064 overlaps with the putative transcription factor binding site (*3′-ATAAA-5′*) for TFIID, which is involved in skeletal bone development and affects *LCORL* (ligand dependent nuclear receptor corepressor like) transcript levels [34]. Lower expression of *LCORL* was observed in horses with the C allele at rs68603064, which was associated with larger animals compared to those with the T allele [34]. This is consistent with the fact that rs68603064 is localized in the orthologous enhancer, a regulatory element that is known to not always affect the nearest promoter but can skip neighboring genes to control genes located further away on a chromosome. In addition, certain enhancers have been shown to regulate multiple genes [35]. For example, a human enhancer ENSR00000718725, located upstream of *LCROL*, regulates four genes: *MED28*, *LCORL*, *piR-32461-120*, and *HSALNG0033171*, according to the GeneCards database [36]. Remarkably, the same polymorphism was also most strongly associated with osteochondritis dissecans in GWAS [37], which should be considered in genetic selection for larger horses. As with GGP_103_BODY_SIZE_ECA11, the marker targets rs68876319 of *LASP1* (LIM and SH3 protein 1), which is also located within an orthologous enhancer. Ponies with the alternative genotypes for rs68876319 (A/G and G/G) have smaller measures for head length, neck length, wither height, croup height, and body length [33]. Two additional markers targeting polymorphic sites in orthologous enhancers within *LASP1* are present on the chip, BIEC2_144152 (rs68875002) and BIEC2_144165 (rs68876315). Among the three markers for *LASP1*, it is interesting to note that rs68875002 has the highest GERP score (0.72); however, no information on this SNP is currently available in the scientific literature. Nevertheless, the genotypes for this marker differ between the P (T|T) and American Miniature (C|C) breeds. Furthermore, comprehensive genome and transcriptome analyses of the small-sized Korean native Jeju horse revealed positive selection for LCORL and LASP1. RNAseq analysis indicated differential expression of these genes, with Jeju horses having a higher proportion of type I muscle fiber (slow twitch) compared to Thoroughbred horses [31]. This also supports the role of LCORL and LASP1 in muscle structure and their potential impact on meat yield and quality traits, as enhancing meat tenderness and quality can be achieved by increasing the proportion of type I muscle fibers [38]. Further studies are needed to validate these markers in different horse breeds.

Three markers were also prioritized for the potential key genes *IGR1R*, *MSTN*, and *PPARGC1A*. According to the bioinformatic analysis, these genes are involved in signal transduction and endocrine system pathways. The GHRH-GH-IGF axis influences the mammalian growth and development [39]. A variant (marker UKUL191) within the *IGF1R* (insulin like growth factor 1 receptor) has been associated with both weight and size in horses [40]. In the present study, an intronic variant rs68514854 (marker BIEC2_44702) within the orthologous regulatory region was prioritized as possibly affecting gene expression (P: G|A, AM: A|A). Expression of *IGF1R* was higher in the lamellar tissue (LT) of lean ponies compared to obese ponies and in LT of ponies fed a low nonstructural carbohydrate (low-NSC) diet compared to ponies fed a high NSC diet. Similarly, more *IGF1R* was measured in the livers of ponies fed a low-NSC diet [41]. In cattle, IGF1R has been demonstrated to be involved in the IGF1R/PI3K/AKT signaling pathway to promote muscle proliferation and differentiation [42], while in dogs, a nonsynonymous variant was associated with smaller size [43]. Furthermore, exercise-induced modifications in skeletal muscle transcriptomes of Arabian horses and the effects of maternal overnutrition on foal muscle development highlight the role of IGF1R in muscle growth, structure, and performance [44,45]. Considering this, it is worth mentioning that there are no markers for the exonic regions of *IGF1R* on the analyzed chip. Anyhow, further studies are needed to establish a link between *IGF1R* polymorphism and meat yield in horses. The *MSTN* (myostatin) gene encodes a protein known to be a negative regulator of muscle mass in mammals [46]. In horses, it has been analyzed for its association with muscularity, strength [46], and race distance [47]. In the present study, we prioritized rs69126368 (marker BIEC2_417365) within the intron five of *MSTN* and with a high GERP score of 3.57 (P: A|G, AM: G|G). Interestingly, this SNP also overlaps with another protein-coding gene *C18H2orf88* (chromosome 2 open reading frame 88). Although little is known about *C18H2orf88*, it is located in a genomic region that distinguishes horse breeds with contrasting risk for degenerative suspensory desmitis [48], a severity that is potentially at risk with increasing body size/weight. It is perhaps noteworthy that the orthologous gene in human is associated with diseases such as muscle hypertrophy and myostatin-related muscle hypertrophy, according to the GeneCards database [36], making the rs69126368 locus an interesting candidate region for future investigation. PPARGC1A (PPARG coactivator 1 alpha) regulates mitochondrial biogenesis and oxidative energy metabolism in muscle and has been recognized as a candidate gene for equine athletic performance [49,50]. Recent research has shown significant exercise-induced changes in *PPARGC1A* expression in equine skeletal muscle, highlighting its role in muscle adaptation and function [51,52]. In cattle, *PPARGC1A* has been identified as one of the key genes associated with intramuscular fat content and reproductive traits [53,54]. These findings suggest that PPARGC1A variants may influence meat yield and quality traits in horses. Here, we have prioritized an intronic variant rs68672779 (marker BIEC2_848001) with a high GERP score of 3.5.

Three more genes have been previously associated with mammalian phenotypes possibly important for horsemeat production: *GULP1*, *SLIT2*, and *RAPH1*. In previous studies, two markers for *GULP1* (GULP PTB domain containing engulfment adaptor 1) have been associated with withers height in racing lines of Quarter Horses; BIEC2_417120 and BIEC2_417135 [55]. For the first marker, animals with the genotype TT were higher than genotypes TC or CC, while for BIEC2_417135, AA genotype was identified as the “favorable” genotype [55]. However, based on our data, both markers target intronic regions without orthologous regulatory elements or high conservation scores. In the present study, we prioritized UKUL3220, which targets a missense variant 18_65173441_T/C (canonical transcript) and the 5 prime UTR of other protein-coding transcripts of *GULP1* within the constrained element and with a very high GERP score of 4.81. Also worth mentioning is the marker CUHSNP00150635, which targets a 3 prime UTR variant 18_65288583_A/C with a GERP score of 3.2. GULP1 positively regulates TGF-β signaling, resulting in cell growth inhibition [56]. Furthermore, up-regulated *Gulp1* expression decreases adipose tissue expansion in mice [57]. This makes *GULP1* and its markers interesting candidates for horsemeat production that should be explored in the future. *SLIT2* (Slit guidance ligand 2) is a gene involved in neuronal development. The gene has been associated with backfat thickness in Landrace and Yorkshire pigs [58] and beef production [59]. In the present study, we prioritized a marker CUHSNP00004551 for a splice donor region variant rs68593800 within the constrained element. As for a newly identified gene in horses, *RAPH1* (ENSECAG00000022376), we prioritized a marker BIEC2_421054 that targets the intronic rs69153424 within a constrained element. The gene is involved in nutrient absorption by rumen epithelial cells [60], possibly via the phosphatidylinositol (3,4) bisphosphate signaling pathway [61]. Interestingly, we also prioritized a marker CUHSNP00004551 for a high-impact stop-lost variant rs1141209077 in *PI4K2B* (phosphatidylinositol 4-kinase type 2 beta), which likely alters protein length and disrupts protein function.

In addition to the above genes, other genes with prioritized markers likely contribute to the desirable traits important for horsemeat production, which will be discussed below. Potential regulatory variants were identified in six other genes: *ADGRA3*, *FAM184B*, *QDPR*, *SEPSECS*, *LDB2*, and *CAST*. The rs68508249 (BIEC2_848365) is located upstream of *ADGRA3* (adhesion G protein-coupled receptor A3) within an orthologous enhancer. These sites are common regions for promoters, agreeing that the orthologous position of rs68508249 also overlaps the mouse and human promoter. ADGRA3/GPR125 participates in cell communication and positively regulates osteoclastogenesis [62], which is involved in bone remodeling and formation that is particularly vigorous during the growth period to increase body size [63]. In mice, reduced osteoclastogenesis led to smaller and lighter animals [64]. Similarly, *ADGRA3* has been associated with body weight in chickens [65]. In addition, the *Adgra3* expression was higher in the adipose tissue of obese mice, and its siRNA-mediated silencing resulted in significantly reduced lipid storage in adipocytes. Therefore, the prioritized SNP may affect body growth and possibly also meat yield and quality.

Another bone-related gene is *FAM184B* (family with sequence similarity 184 member B). Variants within the gene have been associated with height at the withers in German warmblood horses (marker BIEC2_808608) [66] and with body length in Spanish purebred horses (marker BIEC2_808617) [67]. However, a variant rs68534803 (marker BIEC2_808579) in *FAM148B* was associated with osteochondrosis in the tarsocrural joint of Dutch Warmblood and French Trotter horses [68,69]. In the present study, two markers within the orthologous enhancer were prioritized, BIEC2_808625 for rs68454110 (P: C|C, AM: T|T) and BIEC2_808581 for rs68534807, but have not yet been reported in the literature.

In *SEPSECS* (Sep (O-phosphoserine) tRNA:Sec (selenocysteine) tRNA synthase), the prioritized marker BIEC2_808653 targets an SNP rs68520444 (P: A|A, AM: A|G) within the orthologous promoter in the 5 prime UTR and thus has a possible effect on transcription. The gene encodes a protein that participates in selenium metabolism and produces selenocysteine. Selenium primarily plays an important role in immune function and antioxidant capacity [70,71,72]. However, selenoproteins, which contain selenium in the form of selenocysteine, are critical for bone remodeling, and selenium deficiency leads to growth retardation [73]. In cattle, *SEPSECS* is associated with carcass weight [74], so the gene and its polymorphism should be investigated in the future.

Another metabolic gene, *QDPR* (quinoid dihydropteridine reductase), regenerates tetrahydrobiopterin (BH4), which is an essential cofactor for the biosynthesis of catecholamine and serotonin (5-hydroxytryptamine, 5-HT) [75]. BH4 influences the level of phenylalanine. Treatment of patients with phenylketonuria (phenylalanine build-up in the body) with BH4 improves growth, development, and nutrient status [76,77,78]. In chickens, higher expression of *QDPR* was associated with body weight [79]. In the present study, we prioritized an intronic variant rs68520444 (marker BIEC2_808653), which is located within an orthologous enhancer and could therefore affect the expression of *QDPR* and surrounding genes.

The *CAST* (calpastatin) gene is primarily associated with meat quality parameters [80]. In sheep, however, it codes for growth factors, their receptors, and transport and regulatory proteins that contribute to the formation of muscle mass and influence growth rates [81]. The *CAST* gene has not been studied in horses. Recently, however, Belousova et al. (2023) found a polymorphism in the *CAST* of Vyatka horse lines [81]. In chickens, higher expression of *CAST* was found in a commercial meat chicken breed (S01) compared to the black-bone mountain chicken breed (MB) [82], and gene expression was higher in fast-growing rainbow trout than in fish with the slowest growth rate [83]. In the present study, a prioritized marker BIEC2_277089 targets the intronic variant rs68930623, which is located within an orthologous enhancer and potentially affects gene transcription. CAST is an inhibitor of CAPN1 (calpain 1), and together they form a system that regulates skeletal muscle growth, development, and atrophy [84]. In the present study, we also prioritized a marker CUHSNP00133513 targeting a missense variant rs396701927 of *CAPN1*. Considering its location within a constrained element and a very high GERP score of 4.15, this polymorphism is likely to be functionally relevant.

The final marker prioritized for its potential role in regulating gene transcription is BIEC2_808833, which maps to the intronic variant rs68525607 in the orthologous enhancer of *LDB2* (LIM domain binding 2). LDB2 is a transcriptional regulator responsible for the assembly of the transcriptional regulatory complex [85]. In humans, it is associated with neuronal development [86] and the cell cycle [87]. In chickens, however, it was found to be most strongly associated with body weight and late growth [88]. Interestingly, in Hucul mares, *LDB2* was found to be one of the age-related differentially methylated genes in blood cells [89]. In addition to BIEC2_808833, another marker, BIEC2_808856, targeting rs68525653, was prioritized due to its high GERP score (3), making it potentially relevant. Similarly, *LGI2* (leucine rich repeat LGI family member 2) has been suggested to regulate neuron synapse development and maturation [90]. Mice double mutants for *Lgi2/*3 exhibited poor postnatal growth [91]. For *LGI2*, we prioritized a marker UKUL843 targeting a 3 prime UTR variant rs1147560021 with a GERP score of 2.7. Given this, the variant could affect miRNA-mediated *LGI2* regulation (P: C|T, AM: T|T).

Three additional markers were prioritized based on the high GERP score of their polymorphic target sites or their location within a constrained element. These markers were identified in *ABI2* and *PCDH7*. ABI2 (Abl interactor 2), which is involved in actin remodeling and maintenance of cell morphology, has been demonstrated to be under the regulatory control of PRR16/Largen, a cell size regulator that is independent of mTOR and Hippo signaling pathways [92]. In black Bohai cattle, ABI2 has been linked to muscle development [93], while the prioritized marker BIEC2_421048 targeting intronic rs69153418 has been associated with body weight in Japanese Thoroughbred racehorses [94]. Two markers were prioritized for *PCDH7* (protocadherin 7), UKUL834 and UKUL835. Both target missense variants with high GERP scores of 2.83 (rs68555658, P: T|G, AM: G|G) and 3.84 (rs1147724321), respectively. In Simmental beef cattle, *PCDH7* has been proposed as a candidate for regulating body size [95]. The gene may be involved in residual feed intake as suggested in Nelore cattle [96] and feed efficiency in Duroc pigs [97].

Finally, missense variants for *DNAH7* (dynein axonemal heavy chain 7) and *ZNF804A* (zinc finger protein 804A) were prioritized: marker Affx-102281324 for rs396935555 (P: C|T, AM: T|T) in *DNAH7* and marker BIEC2_416678 for rs69192315 in *ZNF804A*. While *DNAH7*, another gene encoding a cytoskeletal protein, is thought to be primarily responsible for fertility [98,99,100], *ZNF804A* regulates pre-messenger RNA processing and gene expression [101]. In cattle, *ZNF804A* has been identified as one of the markers differentiating adipose tissue types, which influences intramuscular fat [102].

## 5. Conclusions

Bioinformatic analysis of the SNP chip helped us to find potentially functional SNP markers for several genes that could be important for horsemeat production, such as *LCORL*, *LASP1*, *IGF1R*, and *MSTN*. This study will benefit smallholder farmers by providing genetic insights for breeding decisions that promote desirable traits for meat production. Identifying specific SNP markers related to body size and weight enables selective breeding for improved meat yield, supporting efficient breeding without extensive genotyping resources. Empirical studies show that targeted weighting improves prediction accuracy, supporting precise and effective breeding decisions [103,104,105,106,107]. The information from this study will be valuable for large-scale genotyping and analyses such as GWAS and GBLUP. Therefore, integrating these prioritized SNP markers with genotypic data from larger horse populations will improve the accuracy of genomic breeding value predictions and support more effective breeding programs aimed at improving meat production, and the principles used in this study could be extended to other economically important traits in horses. Overall, this research not only helps individual breeders and small farmers but also lays the foundation for further development of the broader field of equine genomics. However, this study has some limitations, including reliance on bioinformatics and the literature without experimental validation. In addition, not all traits related to meat yield were examined, such as chest circumference, body length, growth rate, average daily gain, myopathies, muscle mass, number of thoracic vertebrae, and bone strength. Important meat quality parameters, such as fat deposition, fat composition, tenderness, firmness, marbling, and water holding capacity, were also not included. The strict criteria for inclusion or exclusion of SNP markers may also have resulted in relevant SNPs being omitted. In addition, we have focused on canonical transcripts, although other transcripts of the same gene may also play significant roles in meat production traits and should be considered in future analyses. Therefore, future research should experimentally validate these markers, examine a wider range of traits, and include quality parameters to enhance the accuracy of horse breeding schemes using methods such as GWAS and other genomic approaches. Furthermore, it will be essential to assess the variability within individual populations and incorporate this information into breeding programs or provide guidelines for breeders. This approach will ensure that the genetic insights gained from this study are effectively translated into practical breeding strategies.

## Figures and Tables

**Table 1:** Summary of prioritized SNP markers, their associated genes, variant consequences, and the criteria for their prioritization. A ✓ indicates the presence of the respective characteristic: potential effect on protein function, GERP > 2 (indicating evolutionary conservation), location within a constrained element, or location within an orthologous regulatory element. SNPs that differ between Percheron and American Miniature horse breeds are marked in bold.

Gene Symbol	Marker	SNP ID	Variant Consequence	Potential Effect on Protein	GERP > 2	Constrained Element	Regulatory Element	Mammalian Phenotype
*ABI2*	BIEC2_421048	rs69153418	intronic variant			✓		
*ADGRA3*	BIEC2_848365	rs68508249	upstream gene variant				✓	
***C18H2orf88***	**BIEC2_417365**	**rs69126368**	**intronic variant**			**✓**		
*CAPN1*	CUHSNP00133513	rs396701927	missense variant	✓	✓	✓		
*CAST*	BIEC2_277089	rs68930623	intronic variant				✓	
***DNAH7***	**Affx-102281324**	**rs396935555**	**missense variant**	**✓**				
***ENSECAG00000052525***	**GGP_100_BODY_SIZE_ECA3**	**rs68603064**	**intronic variant**				**✓**	
***FAM184B***	**BIEC2_808625**	**rs68454110**	**intronic variant**				**✓**	
*FAM184B*	BIEC2_808581	rs68534807	intronic variant				✓	
*GULP1*	UKUL3220	18_65173441_T/C	missense variant	✓	✓	✓		✓
*GULP1*	CUHSNP00150635	18_65288583_A/C	3 prime UTR variant		✓			
***IGF1R***	**BIEC2-44702**	**rs68514854**	**intronic variant**				**✓**	**✓**
***LASP1***	**BIEC2_144152**	**rs68875002**	**intronic variant**				**✓**	**✓**
***LASP1***	**BIEC2_144165**	**rs68876315**	**intronic variant**				**✓**	
*LASP1*	GGP_103_BODY_SIZE_ECA11	rs68876319	intronic variant				✓	
*LDB2*	BIEC2_808856	rs68525653	intronic variant		✓			
*LDB2*	BIEC2-808833	rs68525607	intronic variant				✓	
***LGI2***	**UKUL843**	**rs1147560021**	**3 prime UTR variant**		**✓**			
***MSTN***	**BIEC2_417365**	**rs69126368**	**intronic variant**			**✓**		**✓**
***PCDH7***	**UKUL834**	**rs68555658**	**missense variant**	**✓**	**✓**			
*PCDH7*	UKUL835	rs1147724321	missense variant	✓	✓			
*PI4K2B*	CUHSNP00004551	rs1141209077	stop-lost	✓				
*PPARGC1A*	BIEC2_848001	rs68672779	intronic variant		✓			✓
***QDPR***	**BIEC2_808653**	**rs68520444**	**intronic variant**				**✓**	
*RAPH1*	BIEC2_421054	rs69153424	intronic variant			✓		✓
*SEPSECS*	BIEC2_806764	rs68670656	5 prime UTR variant				✓	✓
*SLIT2*	CUHSNP00004551	rs68593800	splice donor region variant	✓		✓		✓
*ZNF804A*	BIEC2_416678	rs69192315	missense variant	✓				

## Data Availability

The data that support the findings of this study are available from the corresponding author, A.K., upon reasonable request.

## Supplementary Materials

The following supporting information can be downloaded at: https://www.mdpi.com/article/10.3390/ani14162441/s1, Table S1: Search terms used for screening the PubMed database for the literature related to genetic research for meat production in cattle and horses; Table S2: Detailed map of the 268 SNP markers in genes potentially involved in horsemeat production extracted from the GeneSeek^®^ Genomic Profiler™ Equine 70K SNP Chip; Table S3: Summary of SNP markers identified on the GGP Equine chip within (and 5000 bp up- and down-stream) the genes *CAPN1*, *CAST*, and *MSTN*, which are directly associated with meat yield and quality in cows; Table S4: Summary of QTLs associated with meat production in cattle from Animal QTLdb. These QTLs are categorized into three traits: dressed carcass muscle percentage, dressed carcass muscle weight, and dressed carcass muscle-to-bone ratio; Table S5: Summary of SNP markers on the GGP Equine chip for orthologous genes associated with dressed carcass muscle weight and dressed carcass muscle-to-bone ratio in cattle; Table S6; Summary of SNP markers on the GGP Equine chip for genes associated with body weight in horses; Table S7: Summary of SNP markers on the GGP Equine chip for genes associated with body size in horses; Table S8: Comparison of prioritized SNP markers between Percheron and American Miniature horse breeds; Table S9: Comparison of orthologous genes with prioritized SNPs between horses and cattle.
